# Social media and its relationship with mood, self‐esteem and paranoia in psychosis

**DOI:** 10.1111/acps.12953

**Published:** 2018-09-10

**Authors:** N. Berry, R. Emsley, F. Lobban, S. Bucci

**Affiliations:** ^1^ Division of Psychology and Mental Health School of Health Sciences Faculty of Biology, Medicine and Health Manchester Academic Health Science Centre University of Manchester Manchester; ^2^ Department of Biostatistics and Health Informatics Institute of Psychiatry, Psychology and Neuroscience King's College London London; ^3^ Spectrum Centre for Mental Health Research Lancaster University Lancaster; ^4^ Greater Manchester Mental Health NHS Foundation Trust Manchester UK

**Keywords:** psychosis, schizophrenia, behaviour

## Abstract

**Objective:**

An evidence‐base is emerging indicating detrimental and beneficial effects of social media. Little is known about the impact of social media use on people who experience psychosis.

**Method:**

Forty‐four participants with and without psychosis completed 1084 assessments of social media use, perceived social rank, mood, self‐esteem and paranoia over a 6‐day period using an experience sampling method (ESM).

**Results:**

Social media use predicted low mood, but did not predict self‐esteem and paranoia. Posting about feelings and venting on social media predicted low mood and self‐esteem and high paranoia, whilst posting about daily activities predicted increases in positive affect and self‐esteem and viewing social media newsfeeds predicted reductions in negative affect and paranoia. Perceptions of low social rank when using social media predicted low mood and self‐esteem and high paranoia. The impact of social media use did not differ between participants with and without psychosis; although, experiencing psychosis moderated the relationship between venting and negative affect. Social media use frequency was lower in people with psychosis.

**Conclusion:**

Findings show the potential detrimental impact of social media use for people with and without psychosis. Despite few between‐group differences, overall negative psychological consequences highlight the need to consider use in clinical practice.

**Significant outcomes**

- This is the first study to use ESM to highlight the significant association between self‐reported social media engagement and subsequent reductions in mood at the next time‐point in a sample of individuals with and without experience of psychosis.
- Types of self‐reported behaviours on social media were associated with subsequent changes in mood and paranoia at the next time‐point. Specifically, posting about daily activities was associated with subsequent improvements in positive affect and self‐esteem, whilst viewing social media newsfeeds led to significant reductions in negative affect and paranoia and content consumption was associated with subsequent reduction in negative affect. Conversely, posting about feelings and venting on social media were associated with subsequent reductions in mood and self‐esteem and increases in paranoia and viewing profiles of individuals who were not ‘friends’ on social media and commenting on the posts/pictures of others led to increases in paranoia.
- People with psychosis are significantly less likely to use social media than people without psychosis.

**Limitations**

- The sample was limited to mostly White British participants and clinicians may have been more likely to refer patients who had previously expressed positive or negative experiences of engaging with social media, which may limit the generalisability of the findings.
- The design of the study was correlational in nature, so other factors may have also contributed towards the impact of social media use.
- The study was reliant on participants self‐reporting social media use, which may be subject to inaccurate recall. However, the use of ESM for self‐reported use over a short time‐scale is likely to have helped to mitigate this limitation.

## Introduction

The use of social media websites such as Twitter, Facebook and Instagram is widespread. Social media websites allow individuals to construct profiles in which they can maintain and create social networks, circulate details about their daily lives and respond to posts written by others 1. Rates of social media use by people with severe mental health problems such as psychosis are lower than the general population based on small‐scale studies 2, 3.

People with mental health problems already use social media websites to self‐manage their mental health. For example, social media can be a helpful coping mechanism to facilitate self‐expression and communication with others with similar experiences and to access motivational content 4, 5, 6. Clinicians have observed occasions where online communication has been beneficial for clients' with SMI through accessible peer support and ability for anonymous self‐expression 7. Individuals are amenable to the idea of receiving mental health support via social media websites 8 and have suggested the inclusion of social media components, such as moderated discussion forums, in future interventions 9.

Despite some evidence for the potential therapeutic benefits of social media use, social media engagement may also be harmful for an individual's mental health and wellbeing. For example, several studies have reported a significant link between high social media use and low mood and depression 10, 11, 12. However, others have found no evidence of a link between social media use and mood 13, 14. Mixed findings have also been reported for the relationship between social media use and self‐esteem 15, 16, 17, 18. Systematic reviews have highlighted that conclusions cannot yet be drawn and further robust research is warranted 19, 20. It has been proposed that people with psychosis may be particularly vulnerable to paranoid ideas after using social media websites 21, and evidence from case reports suggests the development and exacerbation of symptoms associated with severe mental health problems after social media engagement 22, 23, 24, 25. Individuals with psychosis may be more affected by content consumption on social media in comparison with those without psychosis due to the posts written by others often being open to individual interpretation. Specifically, people with psychosis can have cognitive biases that can lead them to misinterpret the actions and behaviours of others as threatening or self‐referent. Therefore, a virtual world where one is continuously observing the content written by others may facilitate individuals with psychosis to observe and become suspicious by others actions online. However, much of the current research has relied on participants with severe mental health problems retrospectively self‐reporting whether they feel their use of social media leads to paranoia 3, 22, 25. More recently, Bird et al. 26 reported that the experience of negative affect during social media use correlates with paranoia severity. The lack of robust study designs and larger‐scale research prevents conclusions from being drawn.

Recent findings suggest there may be specific aspects of social media use that determine whether it is beneficial or detrimental. One such explanation is that social media may elicit downward online social comparisons; that is, comparing oneself less favourably to others, leading to negative feelings 27. Festinger 28 highlighted the importance of social comparison to explain the inherent drive for individuals to achieve accurate self‐evaluation of opinions and abilities. Social comparisons lead to the development of social ranks (SRs), whereby individuals compare themselves to others on relative power and social attractiveness 29. Social media may facilitate the formation of SRs due to the tendency for people to present themselves and their experiences in a positive light 30, 31. Researchers working in the field of social comparisons and psychopathology have proposed that perceived SR is associated with mood and self‐esteem 32, 33 and it has previously been reported that negative social comparisons on social media websites are associated with depression and low self‐esteem 34, 35, 36, 37. Indeed, a recent editorial proposed that individuals who experience mental health problems may be more likely to be affected by the social comparisons that social media elicits due to a negative cognitive bias 38.

Researchers have now begun to explore whether social media behaviours contribute to psychological outcomes. Burke et al. 39 separated social media behaviours into (i) directed communication such as posting on another user's profile; (ii) content production such as posting status updates; and (iii) content consumption such as scrolling through social media newsfeeds. Additionally, a recent review highlighted that active social media use can enhance subjective wellbeing, whereas passive social media use reduces subjective wellbeing 40. Therefore, consequences of social media use may relate to social media activities, rather than levels of social media use *per se*.

In order to examine the real‐time relationship between social media use and mental health and wellbeing, some researchers have employed experience sampling methodology. For example, Kross et al. 41 used ESM to demonstrate that Facebook use predicted subsequent declines in subjective wellbeing; although, this effect was not predicted by direct communication on Facebook. Further research used ESM to explore the relationship between passive Facebook use and wellbeing, reporting that envy mediated the relationship between passive Facebook use and declines in affective wellbeing 42. Finally, Jelenchick et al. 14 demonstrated no association between social media use and depression in adolescents through ESM data collection.

## Aims

Previous research has mainly employed retrospective accounts of social media use, SR, mood, self‐esteem and paranoia, with many studies conducted in adolescent non‐clinical samples. This study aimed to explore in real time the impact of social media use on mood, self‐esteem and paranoia. Specifically, we hypothesised:

*H1*: Social media use will predict low mood and self‐esteem and high paranoia.
*H2*: Passive social media use (content consumption) will predict low mood and self‐esteem and high paranoia, whilst active social media use (content posting and direct communication) will predict high mood and self‐esteem and low paranoia.
*H3*: Higher perceived SR in comparison with others on social media will predict high mood and self‐esteem and low paranoia.
*H4:* The impact of self‐reported social media use and behaviours on mood, self‐esteem and paranoia will be moderated by a diagnosis of psychosis, with a stronger relationship evident in the clinical group.

## Method

### Design

Experience sampling method was used to capture momentary assessments of social media use, mood, self‐esteem and paranoia (within‐subjects), and paper‐based questionnaires were used to capture retrospective reports of these variables (between‐subjects). Experience sampling methodology (ESM) was chosen to explore the impact of social media use because it involves the repeated assessment of variables over a specified time‐period, which is more ecologically valid than traditional retrospective measures 43.

### Participants

Participants were recruited via National Health Service (NHS) secondary care mental health services in the UK and through promoting the study on research volunteering websites. Clinical participants were eligible to participate if they had a clinician‐verified experience of first episode psychosis or had received a diagnosis of DSM‐IV schizophrenia‐spectrum disorder or bipolar disorder. Non‐clinical participants were eligible to participate if they self‐reported no current experiences of mental health problems. Further eligibility criteria for all participants were as follows: (i) 18 years of age or over; (ii) able to speak and read English; (iii) able to provide informed consent; (iv) available for a week‐long study; (v) self‐report Facebook or Twitter account; and (vi) self‐report social media use at least three times per week. This paper reports findings from the non‐clinical and psychosis participants only (*n* = 44) due to the small number of participants in the sample with bipolar disorder (*n* = 6). Findings including participants experiencing bipolar disorder are available from the author on request.

### Baseline and trait‐level measures

Trait‐level mood was measured using the Positive and Negative Affect Schedule 44. The PANAS consists of 20 adjectives associated with positive affect (PA) and negative affect (NA) and respondents are asked to indicate their levels of agreement on a 5‐point Likert scale (1 = very slightly or not at all; 5 = extremely). The scale can be used to measure both trait‐ and state‐levels of PA and NA depending on question phrasing 44. The scale has excellent internal consistency for both PA (α = 0.86–0.90) and NA (α = 0.84–0.87). In this study, Cronbach's alpha was 0.90 for PA and 0.91 for NA.

Trait self‐esteem was measured using the Rosenberg Self‐esteem Scale (RSES), which consists of 10 statements on a 4‐point scale (1 = strongly agree; 4 = strongly disagree) and can be used to measure both trait and state self‐esteem 45. The scale has also previously shown excellent internal consistency and test–retest reliability (*r* = 0.85–0.88) 46. In this study, the Cronbach's alpha was 0.95.

Trait paranoia was measured using the Paranoia Scale, which comprises 20 statements on a 5‐point Likert scale (1 = not at all applicable to me; 5 = extremely applicable to me). The scale has been used in both clinical and non‐clinical samples and has shown good construct validity (α = 0.84) and test–retest reliability (*r* = 0.70) 47 and demonstrated excellent internal consistency in this study (α = 0.95).

Baseline perceptions of SR were measured using the Social Comparison Scale (SCS, which consists of a list of 11 pairs of antonyms) 48. Participants are asked to indicate on a 10‐point scale how they feel in comparison with others (e.g. 1 = inferior, 10 = superior). The higher the SCS score, the higher the perceived SR relative to others. The SCS has demonstrated good internal consistency in both clinical (α = 0.91) and non‐clinical (α = 0.88) samples 48 and showed good internal consistency in this study (α = 0.83).

Baseline assessments of social media use were produced after a review of the literature to identify items that had been used in previous studies. The Social Media Use Integration Scale (SMUIS) was included to measure participants' emotional connection to social media and integration into their daily lives 48. The SMUIS is a 10‐item scale ranging from 0 (strongly disagree) to 5 (strongly agree) and has shown strong internal consistency (α = 0.91) and good test–retest reliability (*r* = 0.80) and can be modified for other social media platforms 49. The subsequent six questions focussed on social media privacy settings and were taken from research published by Ross et al. 50. A further 14 questions were adapted from the same questionnaire to assess how often participants reported engagement in certain social media behaviours (eight response options, range: ‘never’ to ‘more than once a day’) and assigned to the overarching activities of content posting and direct communication (active social media use) and content consumption (passive social media use). Social media behaviours were assigned to these overarching categories based on the criteria published by Burke et al. 39. Specifically, the activities of writing status updates (including status updates about daily activities, feelings, opinions and venting) and posting pictures/videos were assigned as content posting; social media newsfeed/timeline scrolls and viewing friends or strangers profiles were assigned as content consumption; commenting on another person's post/picture, liking another person's post/picture and sharing another person's post/picture were assigned as direct communication. These activities were defined and agreed during the development of the ESM items and prior to data collection.

### State‐level (ESM) measures

The first question of the ESM assessments asked participants whether they had used social media since the last assessment. If participants selected ‘yes’, they were asked additional sets of questions regarding social media use prior to completing the remaining ESM assessments: (i) social media website used; (ii) social media activities; and (iii) feelings in comparison with others on social media using the SCS 44. The responses from question 2 were characterised under content posting, direct communication and content consumption also using the criteria developed by Burke et al. 39. Responses for question 3 were combined to produce the SCS scores for each participant at each time‐point. In this study, the momentary assessment of SR using the SCS had a Cronbach's alpha of 0.96.

To account for the potential that other forms of personal interaction may lead to changes in the variables of interest, participants were asked whether they had spoken with another person since the last assessment (yes/no) and to indicate whether this communication was face‐to‐face, online, text‐message, telephone or a messaging smartphone application. Participants were presented with nine mood‐related adjectives on a 7‐point Likert scale 51 and asked the degree to which the adjective described their current feelings (1 = not at all; 7 = very). Responses to negative and positive adjectives were combined for each participant to give a total value for NA and PA at each time‐point and demonstrated excellent internal consistency (α = 0.93; α = 0.94 respectively). The subsequent two questions used four ESM self‐esteem items and four paranoia items 51, 52, 53. For both questions, participants were asked to indicate on a 7‐point Likert scale the extent to which they agreed or disagreed with the items (1 = not at all; 7 = very). Both the state self‐esteem scale (α = 0.96) and state paranoia scale (α = 0.92) demonstrated excellent internal consistency in this study.

A full list of ESM items is available in Appendix S1.

### Procedure

Ethical approval was granted by the local research ethics committee. After consent, participants completed a demographics questionnaire and the baseline and trait‐level measures in order to provide a description of the sample. Participants were given a unique username and password to access the ESM assessments and completed a trial run either on their own smartphone or a smartphone loaned to them for the duration of the study. Participants who were loaned a smartphone for the study were asked to only use the smartphone for activities associated with the study. Specifically, participants were asked to only use the smartphones to contact the researcher with any technical questions that arose during the study period and to complete the momentary assessments. ESM assessments commenced the following morning. Text‐messages containing a link to a secure online site were sent to participants at six pseudo‐random times a day over a 6‐day period. Participants had up to 15 min to click the link and complete the assessments after each text‐message received. The data collection period ranged from 10:00 and 21:00; although, this could be adapted to allow for individual differences in waking hours. Participants were asked to complete an exit evaluation detailing reasons for missed assessments, debriefed by the researcher about the study and provided £20 vouchers (not contingent on the number of assessments completed).

### Data analysis

Trait‐level mood, self‐esteem, paranoia and perceived SR were analysed by comparing between‐group means using independent *t*‐tests in spss Version 22 (IBM Corp., Armonk, NY, USA). ESM data were assessed for normality through visual inspection of histograms and analysis of skewness and kurtosis. Analyses of ESM data were performed using Stata, version 14 (StataCorp, College Station, TX, USA). The hierarchical structure of ESM data (observations are nested within days, within participants) requires multilevel modelling to be used due to the violation of the assumption of independence of observations. Using maximum likelihood, we fitted 3‐level random intercept models a random intercept for each participant, a random intercept for each participant‐day and participant‐beep error term.

To test whether self‐reported social media use (*H1*), social media behaviours (*H2*) and perceived SR (*H3*) predicted mood, self‐esteem and paranoia at the next time‐point, separate multilevel linear regression analyses were estimated with PA, NA, self‐esteem and paranoia as the outcome variables, and self‐reported social media use, behaviours and perceived SR as the predictor variables. In all models, socialisation (whether or not an individual had spoken with another person) and group (clinical and non‐clinical) were included as covariates to ensure any associations with the outcome variables could be attributed to the use of social media, rather than other forms of socialisation or group. To investigate whether the impact of social media use was moderated by a diagnosis of psychosis (*H4*), a further set of multilevel linear regressions were estimated by looking at the two‐way interaction between group and social media use, with employment status and socialisation included as covariates. Finally, whilst we did not make any specific hypotheses relating to between‐group differences in social media use, odds ratios (ORs) and the corresponding 95% confidence intervals were calculated through a multilevel logistic regression to compare the likelihood of social media use between the clinical and non‐clinical groups.

## Results

### Sample characteristics

Fifty‐one people (26 clinical and 25 non‐clinical) consented to participate. Data provided from one clinical participant were excluded from analyses as they did not complete any assessments over the study period. Data from participants with bipolar disorder (*n* = 6) were excluded due to the small sample size. Therefore, the data from a total of 44 participants (25 non‐clinical; 19 psychosis) are reported in this paper. Demographic information is presented in Table 1. The mean age of participants in the psychosis (*M* = 33.7, *SD* = 9.7, range = 22–54) and non‐clinical (*M =* 35.4, *SD =* 14.7, range = 20–62) groups did not significantly differ (t(42) = 0.45, *P* = 0.655). Three participants (16%) in the clinical group and one participant (4%) in the non‐clinical group borrowed a smartphone for the duration of the study. All borrowed smartphones were returned undamaged. Two participants in the clinical group and one participant in the non‐clinical group borrowed a smartphone because they did not own a smartphone; one participant in the clinical group borrowed a smartphone because their own smartphone had poor data connectivity.

**Table 1:** Participant demographic information, clinical characteristics and social media use

Clinical group	*n* (%)	Non‐clinical group	*n* (%)	Test statistic	*P*	Total *n* (%)
Gender		Gender				
Male	7 (37)	Male	11 (44)	χ^2^ = 0.23, df = 1	0.632	18 (41)
Female	12 (63)	Female	14 (56)			26 (59)
Employment status		Employment status				
Working full time	0 (0)	Working full time	13 (52)	χ^2^ = 25.70, df = 4	<0.001	13 (30)
Working part time	2 (11)	Working part time	3 (12)			5 (11)
Working voluntary	2 (11)	Working voluntary	0 (0)			2 (5)
Student	2 (11)	Student	7 (28)			9 (21)
Unemployed	13 (68)	Unemployed	2 (8)			15 (30)
Ethnicity		Ethnicity				
Asian or Asian British	1 (4)	Asian or Asian British	1 (4)	χ^2^ = 2.46, df = 3	0.483	2 (5)
White British	18 (95)	White British	21 (84)			39 (89)
White Other	0 (0)	White other	2 (8)			2 (5)
Mixed Race	0 (0)	Mixed Race	1 (4)			1 (2)
Highest level of education		Highest level of education				
High school	5 (26)	High school	4 (16)	χ^2^ = 9.88, df = 4	0.043	9 (21)
College	9 (47)	College	4 (16)			13 (30)
Some University	3 (16)	Some University	4 (16)			7 (16)
Undergraduate degree	2 (11)	Undergraduate degree	10 (40)			12 (27)
Postgraduate degree	0 (0)	Postgraduate degree	3 (12)			3 (7)
Diagnosis						
First episode psychosis	9 (47)	–		–	–	
Schizophrenia	3 (16)	–		–	–	
Schizoaffective disorder	4 (21)	–		–	–	
Paranoid schizophrenia	2 (11)	–		–	–	
Psychosis NOS	1 (5)	–		–	–	
Social media websites currently used		Social media websites currently used				
Facebook	19 (100)	Facebook	25 (100)		1.000	44 (100)
Twitter	91 (47)	Twitter	15 (60)	χ^2^ = 0.70, df = 1	0.405	24 (55)
Instagram	9 (47)	Instagram	10 (40)	χ^2^ = 0.24, df = 1	0.625	19 (43)
Google+	10 (53)	Google+	6 (24)	χ^2^ = 3.82, df = 1	0.051	16 (37)
Tumblr	3 (16)	Tumblr	1 (4)	χ^2^ = 1.82, df = 1	0.178	4 (9)
Flickr	2 (11)	Flickr	1 (4)	χ^2^ = 0.72 df = 1	0.395	3 (7)
Social media apps currently used		Social media apps currently used				
Facebook	17 (89)	Facebook	23 (92)	χ^2^ = 0.83, df = 1	0.773	40 (91)
Twitter	9 (47)	Twitter	13 (52)	χ^2^ = 0.93, df = 1	0.761	22 (50)
Instagram	8 (42)	Instagram	11 (44)	χ^2^ = 0.16, df = 1	0.900	19 (43)
WhatsApp	11 (58)	WhatsApp	18 (72)	χ^2^ = 0.96, df = 1	0.328	29 (66)
Snapchat	6 (32)	Snapchat	12 (48)	χ^2^ = 1.20, df = 1	0.272	18 (41)
Google+	7 (37)	Google+	5 (20)	χ^2^ = 1.54, df = 1	0.214	12 (27)
Devices used to access social media		Devices used to access social media				
Laptop	8 (42)	Laptop	17 (68)	χ^2^ = 2.95, df = 1	0.086	25 (57)
Desktop	5 (26)	Desktop	10 (40)	χ^2^ = 0.90, df = 1	0.343	15 (34)
Smartphone	18 (95)	Smartphone	23 (92)	χ^2^ = 0.13, df = 1	0.721	41 (93)
Tablet	8 (42)	Tablet	6 (24)	χ^2^ = 1.63, df = 1	0.202	14 (32)

### Completion rates

Out of the total 1584 assessments that were possible during the study period, 1084 were fully completed by participants (*n* = 458 clinical; *n* = 626 non‐clinical; 68.4% response rate). The proportion of assessments completed per participant ranged between 25% and 91.6%. The mean number of completed assessments did not significantly differ between clinical and non‐clinical groups [*t*(42) = 0.63, *P =* 0.532], males and females [*t*(42) = 1.00, *P =* 0.322], on the basis of whether a participant borrowed or owned the smartphone that was used for data entry [*t*(42) = 0.33, *P* = 0.745], employment status (*F*(4, 39)* =* 0.86, *P =* 0.495) or level of education (*F*(4, 39) = 0.22, *P =* 0.928). Finally, there was no correlation between assessment completion rate and age (*r* = −0.64, *P* = 0.678).

It is generally accepted in ESM studies that participants should complete at least a third of assessments for the data to be included in analyses 54. However, this value is arbitrary and given that ESM is not affected by issues relating to missing data, a specific completion rate of assessments is not required 55. One person in the study completed less than the specified value (25%), but the data they provided were still usable for the purpose of this study due to the variations in the timing of the responses and was, therefore, included in all analyses.

### Demographic characteristics and social media use

General social media use across the study duration was not significantly related to participant age (*r* = −0.24, *P* = 0.116), but was associated with mental health status, with participants in the clinical group reporting lower levels of social media use in comparison with participants in the non‐clinical group [*t*(42) = 2.15, *P =* 0.037]. Social media use was not significantly associated with gender [*t*(42) = −1.41, *P* = 0.166], whether a participant borrowed or owned the smartphone that was used for data entry [*t*(42) = 1.17, *P =* 0.249], employment status (*F*(4, 39) = 2.00, *P* = 0.114) or level of education (*F*(4, 42) = 0.64, *P =* 0.638).

### Between‐group differences in trait measures

Trait‐level self‐esteem, perceived SR, mood and paranoia are presented in Table 2. Participants in the non‐clinical group had significantly higher scores for trait‐level self‐esteem and PA in comparison with the clinical group, whilst participants in the clinical group had significantly higher scores for trait‐level NA and paranoia. There were no significant between‐group differences in trait‐level SR scores.

**Table 2:** Participant trait‐level scores of self‐esteem, perceived social rank, positive affect, negative affect and paranoia

Clinical group	*n*	*M*	SD	Non‐clinical group	*n*	*M*	SD	Test statistic	*P*
Self‐esteem	25	12.6	6.1	Self‐esteem	24^a^	22.3	3.5	*t*(41) = 6.5	<0.001
Social rank	25	54.2	16.6	Social rank	25	61.3	9.6	*t*(42) = 1.8	0.079
Positive affect	25	29.5	9.5	Positive affect	25	36.7	4.9	*t*(42) = 3.2	0.002
Negative affect	25	25.6	8.3	Negative affect	25	17.7	7.6	*t*(42) = −3.3	0.002
Paranoia	25	58.6	20.3	Paranoia	25	32.4	10.3	*t*(42) = −5.6	<0.001

a Missing data from 1 participant.

### Does social media use predict subsequent mood, self‐esteem and paranoia?

Table 3 demonstrates that *H1* was partially supported. Social media use negatively predicted PA and positively predicted NA. However, social media use did not predict self‐esteem or paranoia.

**Table 3:** Effect of social media use on positive and negative affect, self‐esteem and paranoia. Effect is unstandardised β coefficient from separate models

Dependent variable	Effect	Standard error	*P*	95% confidence interval
Positive affect	−0.5027	0.2469	0.042	−0.9866, −0.0188
Negative affect	0.5593	0.2628	0.033	0.0442, 1.0744
Self‐esteem	0.0498	0.1783	0.780	−0.2996, 0.3993
Paranoia	0.1272	0.1794	0.478	−0.2245, 0.4789

Multilevel logistic regression analyses were conducted to explore whether trait variables predicted social media use across the study period. Trait‐level PA (β = 0.0021, *SE =* 0.0190, *p =* 0.913, 95% CI [−0.035 to 0.039]), NA (β = 0.0079, *SE =* 0.0173, *P =* 0.646, 95% CI [−0.042 to 0.026]), self‐esteem (β = 0.0018, *SE =* 0.0226, *P =* 0.938, 95% CI [−0.042 to 0.046]), paranoia (β = −0.0127, *SE =* 0.0074, *P =* 0.085, 95% CI [−0.027 to 0.002]) and SR (β = −0.0096, *SE =* 0.0115, *P =* 0.403, 95% CI [−0.032 to 0.013]) did not predict social media use. Therefore, it likely that social media use predicted mood, rather than being predicted by mood.

### Do reported social media behaviours predict subsequent mood, self‐esteem, paranoia and perceived SR?

Table 4 shows that content posting did not predict PA, self‐esteem or perceived SR, but did positively predict NA and paranoia. Content consumption and direct communication were not found to predict PA, self‐esteem, paranoia or perceived SR. However, content consumption did significantly negatively predict NA. Therefore, *H2* was not supported.

**Table 4:** Effect of reported social media behaviours on positive and negative affect, self‐esteem, paranoia and perceived social rank

	*n*	Positive affect		Negative affect		Self‐esteem		Paranoia		Perceived social rank	
		β	SE	β	SE	Β	SE	β	SE	β	SE
*Content posting*	114	0.1148	0.4468	1.2316*	0.4801	0.0499	0.3108	0.6638*	0.3237	−1.1846	1.1983
Posting about daily activities	71	1.3171*	0.5390	0.7508	0.5904	0.9967**	0.3760	0.1629	0.3987	0.7363	1.4666
Posting about opinions	20	−1.082	0.8993	−0.0392	0.9713	−0.2772	0.6235	−0.1005	0.6497	1.6827	2.4000
Posting about feelings	22	−4.8410***	0.8555	4.3416 ***	0.9362	−1.6865**	0.6066	2.7189 ***	0.6279	−7.9489**	2.2059
Posting pictures/videos of self or others	17	0.4534	0.9413	−0.3502	1.0063	−0.1546	0.6485	0.3190	0.6727	−2.3035	2.4867
Venting on social media	13	−4.4309***	1.0676	4.3563***	1.1480	−1.7770*	0.7426	2.1293**	0.7697	−2.8936	2.8688
*Content consumption*	514	0.1816	0.4710	−1.1180*	0.4993	0.5389	0.3251	−0.5269	0.3346	1.7166	1.2481
Looking through newsfeed	470	0.2280	0.4444	−1.0748*	0.4720	0.5788	0.3078	−0.8823**	0.3148	1.9190	1.1773
Viewing friends' profiles	121	0.5025	0.4346	−0.0271	0.4672	−0.1166	0.3012	−0.1016	0.3133	2.7343*	1.1505
Viewing profiles of people who are not friends	47	−0.7552	0.6107	0.6353	0.6549	−0.4298	0.4219	1.2631**	0.4349	−2.9803	1.6407
*Direct communication*	166	−0.0148	0.3787	0.1451	0.4069	−0.0255	0.2624	0.4852	0.2716	0.9634	1.0078
Commented on another person's post/picture	113	−0.0416	0.4315	0.0571	0.4649	−0.0511	0.2993	0.6584*	0.3102	1.0097	1.1515
Liked another person's post/picture	43	−0.1700	0.7014	0.2100	0.7531	−0.0748	0.4871	0.0126	0.5041	1.0298	1.8664
Shared another person's post/picture	32	−0.2047	0.7112	−0.1488	0.7612	−0.0489	0.4900	0.2866	0.5099	−1.9830	1.8829

***P < 0.001, **P < 0.01, *P < 0.05.

Posting about daily activities led to increases in PA and self‐esteem at the next time‐point. Conversely, posting about feelings led to subsequent increases in NA and paranoia and reductions in PA, self‐esteem and perceived SR; venting on social media negatively predicted PA and self‐esteem and positively predicted NA and paranoia; looking through social media newsfeeds negatively predicted NA and paranoia; viewing a ‘friends’ social media profile positively predicted perceived SR; viewing profiles of people who were not ‘friends’ on social media positively predicted paranoia; and commenting on other peoples' statuses positively predicted paranoia.

### Does perceived SR when using social media predict subsequent mood, self‐esteem and paranoia?

Table 5 shows that higher perceived SR when using social media positively predicted PA and self‐esteem and negatively predicted NA and paranoia. These findings support *H3* and demonstrate that perceptions of high SR when using social media predict subsequent increases in mood and self‐esteem and decreases in paranoia.

**Table 5:** Effect of perceived social rank on positive affect, self‐esteem, negative affect and paranoia. Effect is unstandardised β coefficient

Dependent variable	Effect	Standard error	*P*	95% confidence interval
Positive affect	0.1232	0.0132	<0.001	0.0976, 0.1488
Self‐esteem	0.0901	0.0098	<0.001	0.0710, 0.1092
Negative affect	−0.1186	0.0152	<0.001	−0.1484, −0.0888
Paranoia	−0.0780	0.0105	<0.001	−0.0987, −0.0574

### Are lower scores for positive affect and higher scores for negative affect after social media use moderated by experiencing psychosis?

Psychosis was not found to moderate the relationship between social media use and PA (β = 0.1974, *SE* = 0.4969, *P* = 0.691, 95% CI [−0.776 to 1.171]) or NA (β = −0.0876, *SE* = 0.5291, *P =* 0.869, 95% CI [−1.125 to 0.949]). This finding does not support *H4* that psychosis would moderate associations between social media use and mood.

We also explored whether psychosis moderated the relationship between specific types of social media use that had led to significant changes in outcome variables. Psychosis did not moderate the relationship between: (i) content posting and NA (β = 0.7558, *SE* =0.9746, *P* = 0.438, CI [−1.154 to 2.666]) or paranoia (β = −0.0882, *SE* = 0.6580, *P =* 0.894, CI [−1.380 to 1.203]); (ii) posting about daily activities and PA (β = 0.7114, *SE* = 1.0824, *P =* 0.511, CI [−1.410 to 2.833]) or self‐esteem (β = 1.1927, *SE =* 0.7537, *P =* 0.114, CI [−0.284 to 2.670]); (iii) posting about feelings and PA (β = −1.0790, *SE =* 1.7409, *P =* 0.535, CI [−4.491 to 2.333]), NA (β = 3.2388, *SE =* 1.8877, *P =* 0.086, CI [−0.461 to 6.939]), self‐esteem (β = −0.7087, *SE =* 1.2316, *P =* 0.565, CI [−3.123 to 1.705]), paranoia (β = −1.1283, *SE* = 1.2722, *P* = 0.375, CI [−3.622 to 1.365]) or perceived SR (β = 3.1986, *SE =* 4.7332, *P =* 0.499, CI [−6.078 to 12.476]); (iv) venting on social media and PA (β = −2.3189, *SE* = 2.1563, *P* = 0.282, CI [−6.545 to 1.907]), self‐esteem (β = 0.4254, *SE* = 1.5018, *P* = 0.777, CI [−2.518 to 3.369]) or paranoia (β = −0.6628, *SE* = 1.5572, *P =* 0.670, CI [−3.715 to 2.389]); (v) content consumption and NA (β = −1.2609, *SE =* 1.0988, *P =* 0.251, CI [−3.414 to 0.893]); (vi) viewing social media newsfeeds and NA (β = 0.4351, *SE* = 1.0026, *P =* 0.664, CI [−1.530 to 2.40]) or paranoia (β = −0.0764, *SE =* 0.6698, *P =* 0.909, CI [−1.389 to 1.236]); (vii) viewing profiles of friends and perceived SR (β = 0.8748, *SE =* 2.3684, *P =* 0.714, CI [−3.802 to 5.552]); vii) viewing profiles of strangers and paranoia (β = −1.3931, *SE* = 0.8995, *P* = 0.121, CI [−3.156 to 0.370]); and (viii) commenting on another person's post or picture (β = 0.7896, *SE =* 0.6675, *P =* 0.237, CI [−0.519 to 2.098]. However, the relationship between venting on social media and subsequent NA was significantly moderated by psychosis (β = 4.7100, *SE =* 2.3101, *P =* 0.041, CI [0.182–9.238]).

### Do social media use and behaviours differ between people with and without psychosis?

Clinical participants were less likely to use social media than non‐clinical participants (OR = 0.5366, *SE* = 0.1550, *P* = 0.031, 95% CI [0.305–0.945]). Separate analyses revealed that clinical participants were less likely to use Facebook (OR = 0.5394, *SE* = 0.1692, *P* = 0.049, CI [0.292–0.998]), but there were no differences in Twitter (OR = 1.1484, *SE*=0.9881, *P* = 0.871, 95% CI [0.213–6.201]) or Instagram (OR = 0.6580, *SE*=0.7151, *P* = 0.700, 95% CI [0.078–5.536]) use.

## Discussion

This study aimed to identify whether social media use predicted subsequent mood, self‐esteem and paranoia, pinpointing any specific social media behaviours reported by participants that contributed towards relationships observed. Additionally, we aimed to determine whether perceptions of SR when using social media predicted these outcomes and to ascertain whether experiencing psychosis moderated any relationship between social media use and mood, self‐esteem and paranoia.

As hypothesised, social media use predicted reductions in PA and elevations in NA. However, contrary to expectations, social media use was not found to predict self‐esteem or paranoia. Further analyses revealed that posting about daily activities predicted high PA and self‐esteem, whereas posting about feelings and venting on social media predicted low PA, self‐esteem and perceived SR and high NA and paranoia. Seidman (2013) proposed that posting about feelings and venting on social media is an emotional form of self‐disclosure, whereas posting information about daily activities is general self‐disclosure 56. Emotional self‐disclosures are important for social connectedness, belonging and feelings of intimacy 57, 58, 59 and people perceive personal disclosures on social media as a helpful mechanism to connect with others, maintain relationships, exchange opinions and receive support 60, 61, 62. However, in contrast to these findings, it was general factual‐based disclosures that were beneficial for increasing mood, whilst emotional‐based posting was detrimental to mood and paranoia. One tentative explanation for this unexpected finding is that participants did not receive the supportive and reinforcing responses they hoped for when they posted emotional self‐disclosures. This possibility is supported by recent research that found social attraction towards social media users was lower when posts contained highly personal or negative self‐disclosures 63 and that negative online disclosures by individuals with low self‐esteem receive undesirable responses due to the negativity expressed 64.

In contrast with previous research 37, 40, content consumption was found to lead to decreases in NA and there was no relationship between content consumption and perceived SR whilst engaging with social media. The discrepancy in findings between this study and previous studies may be linked to the recent changes in content that individuals observe on social media and, in particular, Facebook. Specifically, it has been widely reported that social media websites now contain a disproportionate number of advertisements and news articles in comparison with actual status updates and posts from social media friends. Indeed, a recent announcement by Facebook in 2018 highlighted the evident reduction in the presence of personal posts on the platform due to the plethora of posts from business and the media, resulting in the aim to reduce this public content 65. Therefore, social media consumption may have been less likely to elicit comparative self‐reflections than previous studies due to the reduced exposure to others lives prevalent in the past. The evolving nature of social media platforms means that future research should examine the impact of the upcoming change in website content.

Experiencing psychosis did not moderate the impact of social media use or behaviours; however, this did moderate the relationship between venting and NA. This suggests that maladaptive coping strategies, such as venting, may be more detrimental for individuals who experience psychosis in comparison with the general population. Although, on the whole, a diagnosis of psychosis did not moderate the impact of social media use and behaviours, the finding remains clinically important because the impact of social media use may have more serious consequences in individuals with psychosis due to lower mood prior to engagement. Therefore, the observed impact of social media use warrants further consideration in the context of psychosis and mental health and wellbeing more generally. Additionally, clinical participants were significantly less likely to use social media than non‐clinical participants. Small‐scale studies demonstrate lower social media website access by individuals with severe mental health problems than the general population 2, 3; however, this is the first to identify differences in frequency of use. Higher levels of paranoia were also associated with lower levels of social media use and, given that the clinical group showed significantly higher scores for both trait‐ and state‐level paranoia, it is likely that the experience of paranoia led to lower levels of social media use. Other potential reasons for these findings should be explored in future studies to determine whether differences in social media use frequency are due to barriers to use or other factors such as symptom occurrence or avoiding social media to prevent any associated negative outcomes.

### Strengths, limitations and directions for future research

A strength of the current study was the ecologically valid prospective measurement of social media use and psychological outcomes. Additionally, socialisation was included to identify whether other forms of social interaction may have predicted outcomes. Trait SR, mood, self‐esteem and paranoia were also measured at baseline and were not found to predict social media use. Therefore, although exact directions cannot be established, the inclusion of these trait measures improves confidence in conclusions regarding outcomes associated with social media use. Comparison of clinical and non‐clinical groups allowed the between‐group comparison of the impact of social media use.

We decided not to actively track participants' social media use due to (i) the costs associated with the passive monitoring of accounts; (ii) concerns that seeking permission to view social media accounts would affect recruitment rates; therefore, negatively impacting the sample size; (iii) the potential for risk identification on accounts and the resulting responsibility for the researcher to report such information; (iv) the potential for participants to change their behaviours on social media if they are aware that a researcher is monitoring their profiles; (v) monitoring would only allow the identification of content posting behaviours; not content consumption; and (vi) accessing participant social media profiles would enable the identification of posts written by others on the individual's profile, who will not have provided consent for researchers to view their posts. ESM was used to reduce the likelihood of inaccurate retrospective recall due to the momentary nature of alerts; however, monitoring participants' social media access and behaviours may have produced more reliable data due to the self‐reported nature of the study. Additionally, we speculated that the detrimental impact of emotional self‐disclosures may be related to the feedback individuals received; however, without clear knowledge of user responses to posts, firm conclusions cannot be drawn. Therefore, future research should seek to identify the responses to such disclosures to understand whether response type mediates the relationship between disclosure posting and outcome. It is also possible that the information participants were presented with when viewing social media profiles and newsfeeds may have contributed towards reductions in mood when using social media. Participants were not asked to provide details about the information they saw each time they accessed social media sites. Future research should expand on these findings by exploring whether type of content viewed contributes towards the impact of use and behaviours. The sample was limited to mostly White British participants so findings are unlikely to be generalisable. Moreover, clinicians may have been more likely to refer patients who had previously described positive or negative experiences of engaging with social media. The design of the study was correlational in nature and it is likely that other factors may have also contributed towards the impact of social media use. Therefore, future research should seek to experimentally manipulate social media use to explore whether the effect is directly attributable specifically to social media use and behaviours. Finally, a diagnosis of SMI was not found to moderate the relationship between social media use and mood; however, the sample size may have not been sufficient for moderation. Therefore, future research should explore the impact of an SMI diagnosis with a larger pool of participants.

### Clinical implications

Despite finding that psychosis did not moderate the impact of social media use per se, reductions in mood after social media use are likely to be more damaging for people with psychosis due to reporting lower levels of mood initially. The negative consequence of social media engagement in both groups highlights the importance of continued consideration of the impact of social media in mental health settings. Specifically, clinicians should ensure that they are aware of and explore any potential issues clients face when using social media, particularly with regard to online self‐disclosures. Additionally, the findings support the recent assertion made by the Royal Society for Public Health (2017) that social media websites should be used to discretely reach out to individuals who may be affected by content and signpost appropriate support options 66. Social networking components such as forums could be incorporated into digital health interventions for severe mental health problems to connect people with similar experiences and provide professional and peer support 67, 68, 69, 70, 71, 72. However, our findings suggest that emotional disclosures via these platforms have the potential to elicit negative feelings. We speculated that the impact of such disclosures may be due to the absence of supportive feedback; therefore, these social networking components should be moderated to ensure individuals receive supportive responses when self‐disclosing personal information. Finally, we identified significantly lower frequencies of social media use by people who experience psychosis, which may be a potential barrier in the uptake of social networking components within DHIs. Further research is needed to explore the reasons for this comparatively low use and to identify whether these differences exist on a larger‐scale.

## Supporting information


**Appendix S1.** ESM assessments.
