# Supplementary material for: Social media and its relationship with mood, self‐esteem and paranoia in psychosis
Source: Acta Psychiatr Scand. 2018 Sep 10;138(6):558–70. doi: 10.1111/acps.12953 (PMC6221086; doi:10.1111/acps.12953)
Supplement: Supplementary file 1 — Appendix S1. ESM assessments. [file ACPS-138-558-s001.docx]

**ESM Assessments**

**Version 2: 01/09/16**

1. Have you been on social media since the last text alert?

Yes

No

If yes branch to:

2. Which social media site did you use? *(please select all that apply)*

Facebook

Twitter

Instagram

Other, please specify ­­­­­­____________

3. What did you do when you last used social media? (*please select all that apply*)

Posted a status/tweet about my daily goings on

Posted a status/tweet about how I was feeling

Posted a “selfie”

Posted a status/tweet about my mental health

Used social media to vent

Expressed my opinion on social media

Commented on another person’s status/tweet/picture

Clicked the “like” button on another person’s status/tweet/picture

Shared another person’s status/tweet

Looked through my Facebook/Twitter/Instagram newsfeed

Looked at a Facebook/Twitter/Instragram’s friend’s profile

Looked at the Facebook/Twitter/Instagram profile of someone who is not a

friend/follower on social media.

Other, please specify ________________

4. *(if participant has indicated social media use – if not skip to question 5).*

Please indicate how you felt about yourself in comparison to others when you last used social media:

| 4.a. Inferior | 1 2 3 4 5 6 7 8 9 10 | Superior |
| --- | --- | --- |
| 4.b. Incompetent | 1 2 3 4 5 6 7 8 9 10 | Competent |
| 4.c. Unlikeable | 1 2 3 4 5 6 7 8 9 10 | Likeable |
| 4.d. Left out | 1 2 3 4 5 6 7 8 9 10 | Accepted |
| 4.e. Different | 1 2 3 4 5 6 7 8 9 10 | Similar |
| 4.f. Untalented | 1 2 3 4 5 6 7 8 9 10 | More talented |
| 4.g. Weaker | 1 2 3 4 5 6 7 8 9 10 | Stronger |
| 4.h. Unconfident | 1 2 3 4 5 6 7 8 9 10 | Confident |
| 4.i. Undesirable | 1 2 3 4 5 6 7 8 9 10 | More desirable |
| 4.j. Unattractive | 1 2 3 4 5 6 7 8 9 10 | More attractive |
| 4.k. An outsider | 1 2 3 4 5 6 7 8 9 10 | An insider |

5.a. Since the last text alert, have you spoken to another person (*e.g. face-to-face, online, telephone, text message*)?

Yes

No

If yes branch to:

5.b. When you spoke to another person since the last text alert, was this face-to-face, online, text message, via telephone or on a messaging app (e.g. Whatsapp)? (*please select all that apply*)

Face-to-face

Online

Text

Telephone

Messaging app (e.g. Whatsapp)

6. Please indicate to what extent you **currently** feel (1 = not at all; 7 = very):

| 6.a. Down | 1 2 3 4 5 6 7 |
| --- | --- |
| 6.b. Lonely | 1 2 3 4 5 6 7 |
| 6.c. Cheerful | 1 2 3 4 5 6 7 |
| 6.d. Anxious | 1 2 3 4 5 6 7 |
| 6.e. Satisfied | 1 2 3 4 5 6 7 |
| 6.f. Relaxed | 1 2 3 4 5 6 7 |
| 6.g. Guilty | 1 2 3 4 5 6 7 |
| 6.h. Insecure | 1 2 3 4 5 6 7 |
| 6.i. Happy | 1 2 3 4 5 6 7 |

7. Please indicate to what extent you agree with the following statements about yourself **at the moment** (1 = not at all; 7 = extremely):

| 7.a. I am a good person | 1 2 3 4 5 6 7 |
| --- | --- |
| 7.b. I am a success | 1 2 3 4 5 6 7 |
| 7.c. I am proud of myself | 1 2 3 4 5 6 7 |
| 7.d. I like myself | 1 2 3 4 5 6 7 |

8. Please indicate to what extent you agree with the following statements about others **at the moment** (1 = not at all; 7 = extremely):

| 8.a. I feel that others dislike me | 1 2 3 4 5 6 7 |
| --- | --- |
| 8.b. I feel that others might hurt me | 1 2 3 4 5 6 7 |
| 8.c. I feel suspicious | 1 2 3 4 5 6 7 |
| 8.d. I feel safe | 1 2 3 4 5 6 7 |
